# Genetic Characterization of Seoul Virus in the Seaport of Cotonou, Benin

**DOI:** 10.3201/eid2710.210268

**Published:** 2021-10

**Authors:** Guillaume Castel, Ravi Kant, Sylvestre Badou, Jonas Etougbétché, Henri-Joël Dossou, Philippe Gauthier, Gualbert Houéménou, Teemu Smura, Tarja Sironen, Gauthier Dobigny

**Affiliations:** CBGP, INRAE, CIRAD, IRD, Institut Agro, Univ Montpellier, Montpellier, France (G. Castel, S. Badou, J. Etougbétché, H.-J. Dossou, P. Gauthier, G. Dobigny);; University of Helsinki, Haartmaninkatu 3, Helsinki FI-00290, Finland (R. Kant, T. Smura, T. Sironen); U; niversity of Helsinki, Agnes Sjöbergin katu 2, Helsinki FI-00790 (R. Kant, T. Sironen);; Laboratoire de Recherche en Biologie Appliquée, Unité de Recherche sur les Invasions Biologiques, Ecole Polytechnique d'Abomey-Calavi, Université d'Abomey-Calavi, Cotonou, Benin (S. Badou, HJ. Dossou, J. Etougbétché, G. Houéménou);; Laboratoire de Génétique Moléculaire et d'Analyse des Génomes, Faculté des Sciences et Techniques,Université d'Abomey-Calavi, Cotonou (S. Badou);; Institut de Géographie, d'Aménagement du Territoire et d'Environnement, Université d'Abomey-Calavi, Cotonou (H.-J. Dossou).

**Keywords:** Seoul orthohantavirus, respiratory infections, SEOV, Rattus norvegicus, rats, rodents, zoonoses, trade, seaport, Cotonou, Benin, Africa, Seoul virus, viruses

## Abstract

Seoul virus is a zoonotic pathogen carried by the brown rat *Rattus norvegicus*. Information on its circulation in Africa is limited. In this study, the virus was detected in 37.5% of brown rats captured in the Autonomous Port of Cotonou, Benin. Phylogenetic analyses place this virus in Seoul virus lineage 7.

Rodents are the most diversified order of wild mammals and are also the prevailing mammal lineage associated with human-inhabited socioecosystems. In addition to their destructive behaviors, rodents are involved in maintaining, disseminating, and transmitting zoonotic pathogens, impacting both animal and human health (*1*).

Hantaviruses are transmitted to humans by inhalation of aerosols contaminated by rodent excreta, including urine, feces, and saliva. They are the causative agents of hemorrhagic fevers and are considered emerging pathogens that impact public health worldwide. Hantaviruses are enveloped with a tripartite single-stranded RNA genome of negative polarity comprising small (S), medium (M), and large (L) segments.

Because hantaviruses are host-specific, their geographic distribution is tightly linked to that of their host. However, the emergence of hantaviruses in new geographic regions is still possible by the spread of the rodent reservoir (*2*). Transport-mediated dissemination of rodent-borne hantaviruses is of critical importance in their distribution and constitutes a critical health concern (*3*). Seoul virus (SEOV), an orthohantavirus first identified in South Korea in 1982, has had a particular impact on global human health attributable to its worldwide dispersal (*2**,**4*). Some outbreaks are hypothesized to be driven by the sporadic introduction of its now cosmopolitan host, the Norway or brown rat (*Rattus norvegicus*), at seaports or from pet and laboratory rats (*5**,**6*). Little is known concerning the circulation of SEOV in Africa, although a recent study reported its presence in southeastern Senegal (*7*). We screened rats in the Autonomous Port of Cotonou, Benin, to determine the presence of SEOV in these rodents.

## The Study

We trapped rodents in the seaport of Cotonou using Sherman line capture traps and locally made wide mesh traps that were set for 3 consecutive nights in April 2018. We transported the animals to the Laboratoire de Recherche en Biologie Appliquée laboratory in closed containers and processed them the same day. We anesthetized the rodents with diethyl ether and subsequently euthanized them by cervical dislocation. 

We screened blood samples from 32 brown rats and 37 house mice (*Mus musculus*) for the presence of hantavirus-reactive antibodies by using an immunofluorescence assay as previously described (*8*). A total of 12 (37.5%) rats were seropositive, and the lungs of 2 rats were confirmed positive by reverse transcription PCR as previously described (*9*). This discrepancy cannot be explained by the presence of maternal antibodies because all seropositive rats were adults except for 1 subadult. However, the difference could be because of the limited sensitivity of the panhantavirus PCR or to a viral load decrease over time that fell below detection limits in rats that were infected several months earlier.

We treated reverse transcription PCR–positive samples with DNase I (Thermo Fisher Scientific, https://www.thermofisher.com), and purified samples with Agencourt RNA Clean XP magnetic beads (Beckman Life Sciences, https://www.beckmancoulter.com). We removed ribosomal RNA using a NEBNext rRNA depletion kit (New England BioLabs, https://www.neb.com). We prepared the sequencing library with a NEBNext Ultra II RNA library prep kit and quantified it using a NEBNext Library Quant kit for Illumina (Illumina, https://www.illumina.com). We sequenced pooled libraries on a MiSeq platform using a MiSeq v3 reagent kit with 300 bp paired-end reads. Raw sequence reads were trimmed and low-quality (quality score <15) or short (<36 nt) sequences were removed using Trimmomatic (*10*). The trimmed sequence reads were assembled against reference sequences (GenBank accession nos. NC_005237.1, NC_005236.1, NC_005238.1) using Bowtie2 algorithm (*11*) and some in-house scripts.

We deposited S, M, and L segment sequences of strain Benin1368 into GenBank (accession nos. MW561221–3) and analyzed them by using BLAST (https://blast.ncbi.nlm.nih.gov). BLAST revealed that the best matches were with SEOV strain CSG5 (accession nos. AB618112–30) from Vietnam for the S segment (97.51% nt identity) and the M segment (97.70% nt identity) and with SEOV strain Lyon/Rn/FRA/2013/LYO852 (accession no. KF387723) from France for the L segment (96.82% nt identity).

We performed phylogenetic analyses on 3 datasets composed of complete or nearly complete coding regions of S, M, and L segment sequences of SEOV from different geographic areas available in GenBank. We used sequences of Hantaan and Anjozorobe viruses as outgroups in all analyses. We performed phylogenetic analyses as previously described (*12*) using the general time-reversible plus gamma distribution plus invariant sites model (S and M segment) or the general time-reversible plus gamma distribution model (L segment).

**Figure 1:**
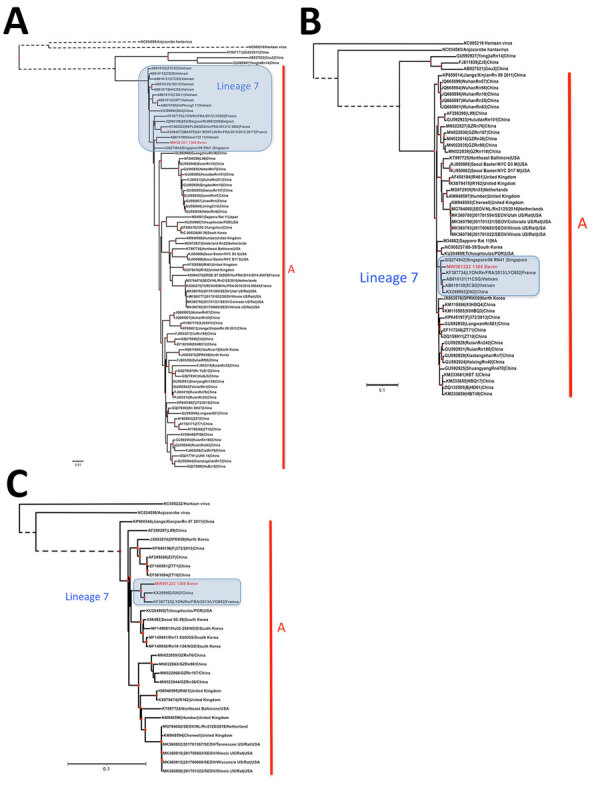
Phylogenetic analysis of Seoul virus complete gene segments recovered from brown rats (*Rattus norvegicus* [family Muridae, subfamily Murinae]) trapped in Benin (red) and reference sequences. Phylogenetic trees were generated by the maximum-likelihood method on the complete coding region of the small (A), medium (B) and large (C) segments. Red points at each node represent branch support with probabilities >75% as determined by an approximate likelihood ratio test. Lineage 7 and phylogroup A are indicated. GenBank accession numbers are provided for reference sequences. Dashed branches have been cut to improve figure readability and are not to scale. Scale bars indicate numbers of substitutions per nucleotide.

The 3 datasets produced broadly concordant phylogenetic topologies (Figure 1). All datasets grouped the SEOV strains from Cotonou within a cluster that included strains from Europe (France and Belgium) and from Southeast Asia (Indonesia, Singapore, Vietnam, and Cambodia), referred to as SEOV lineage 7 (*13*). Variants of this lineage belonged to SEOV phylogroup A; this group originated in China and subsequently spread to other parts of the world (*2*). More specifically, SEOV lineage 7 may reflect the historical connections between regions of Southeast Asia and France through critical trade routes (*14*).

**Figure 2:**
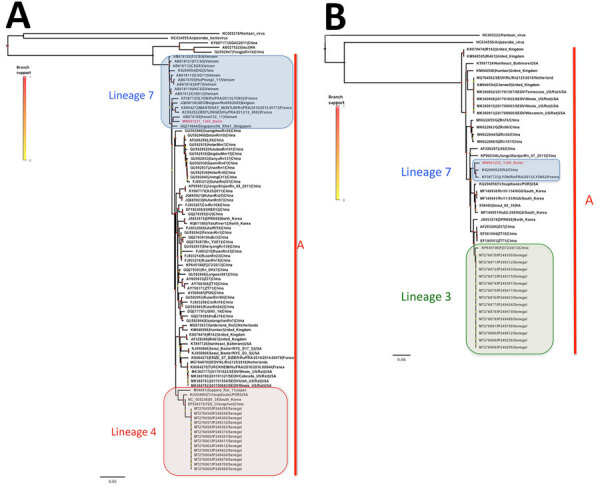
Phylogenetic analysis of Seoul virus partial gene fragments recovered from brown rats (*Rattus norvegicus* [family Muridae, subfamily Murinae]) trapped in Benin (red) and reference sequences. Phylogenetic trees were generated by the maximum-likelihood method on partial coding regions of the small (A) and large (B) segments; medium segments were not done because they were not available from the reference genes from Senegal. Colored points at each node represent branch support as determined by an approximate likelihood ratio test. Lineages 7, 4, and 3 and phylogroup A are indicated. GenBank accession numbers are provided for reference sequences. Scale bars indicate numbers of substitutions per nucleotide.

From Africa, only short SEOV sequences from conserved parts of S (226 nt) and L (347 nt) segments were available from wild black rats (*R. rattus*) from Senegal (*7*). For this reason, we did not include them in our datasets using complete segments. However, phylogenetic analyses on the basis of datasets including these short sequences place them in SEOV lineage 4 (S segment) or 3 (L segment), with low branch support (Figure 2). This inconsistency is potentially attributable to the short length and high conservation of these sequences; although it could indicate a distinct introduction event from Benin, this interpretation must be considered with caution because of the low level of phylogenetic information provided by these sequences. We calculated estimates of evolutionary divergence between strains from Senegal and Benin using MEGAX (http://www.megasoftware.net). Analyses showed 95.7% nt homology for the S segment and 94.8% nt homology for the L segment. Analyses showed 97.85% amino acid-level homology for the nucleocapsid protein and 100% amino acid-level homology for the RNA polymerase.

## Conclusion

Because of insufficient testing for hantavirus infections and unreported mild cases (*14*), the exact circulation of hantaviruses on the continent of Africa is unknown. Whereas SEOV is not widely considered a public health issue in Africa by local health authorities, the presence of SEOV-like agents in humans and wild rats is strongly suspected in at least 17 different countries (*4*). Recent and unambiguous sequencing-based identification of SEOV in Senegal (*7*) and in Benin with our study confirms that SEOV should be anticipated as a possible cause of illness, such as hemorrhagic fever with renal syndrome. Seaports and ships have already been identified as potential entry points for hantaviruses (*2**,**15*), which is a likely cause in Africa, as our study shows. SEOV strains recovered from brown rats from the Cotonou seaport are phylogenetically similar to strains from Southeast Asia and Europe, regions where many maritime trade exchanges occur that could explain the presence of these strains in Cotonou. The accidental transportation of SEOV-carrying rats at seaports could lead to local emergence of SEOV infections among port workers. Regular sanitary control of rats within seaports could prevent rodentborne and arthropodborne pathogen dissemination through sea trade.
